# An unusual association between HIV and Creutzfeldt-Jakob disease in a patient from northeastern Brazil

**DOI:** 10.1016/j.bjid.2025.104590

**Published:** 2025-10-11

**Authors:** José Wagner Leonel Tavares-Júnior, Francisco José Basílio, Francisco Edson Buhamra Abreu, Lucas Rodrigues Tomaz dos Santos, Pablo Picasso de Araújo Coimbra, Érico Antonio Gomes de Arruda

**Affiliations:** a Universidade Federal do Ceará, Departamento de Medicina Clínica, Fortaleza, CE, Brazil; b Hospital São José de Doenças Infecciosas, Fortaleza, CE, Brazil; c Universidade Estadual do Ceará, Fortaleza, CE, Brazil; d Centro Universitário Christus (Unichristus), Fortaleza, CE, Brazil

**Keywords:** Prion diseases, Creutzfeldt-Jakob disease, Rapidly progressive dementia, HIV infection

## Abstract

Prion diseases are significant contributors to rapidly progressive dementia. Among these conditions, sporadic Creutzfeldt-Jakob Disease (CJD) is the most prevalent, characterized by its rarity, lack of treatment options, and rapid progression to fatality. Diagnosis relies on a combination of clinical symptoms and specific alterations detected in brain MRI, EEG, and CSF analysis. The present study details the case of a 53-year-old individual from Fortaleza, Brazil, diagnosed with sporadic CJD, confirmed through clinical presentation and a series of diagnostic evaluations, including 14-3-3 protein detection and RT-QuIC analysis. Differential diagnoses were considered to rule out other rapidly progressing conditions, such as infectious and immune-related diseases, ultimately leading to a likely diagnosis of sporadic CJD.

## Introduction

HIV-Associated Neurocognitive Disorder (HAND) remains a significant challenge in People Living With HIV (PLWH), despite the effectiveness of combined Antiretroviral Therapy (cART), which has made HIV a manageable and treatable disease. The early infiltration of HIV into the Central Nervous System (CNS), loss of blood-brain barrier integrity, activation of immune cells and neuroinflammation, and co-infection with both chronic and acute pathogens are some of the factors involved in its pathogenesis. Clinical manifestations of HAND are classified into three degrees of severity: Asymptomatic Neurocognitive Impairment (ANI), Mild Neurocognitive Impairment (MNI), and HIV-Associated Dementia (HAD), the most severe form, characterized by profound motor and memory deficits that typically result in death^1^.

Creutzfeldt-Jakob Disease (CJD) is a primary form of prion disease, and a major cause of Rapidly Progressive Dementia (RPD), an uncurable condition. CJD includes sporadic, familial, variant, and iatrogenic forms^2^, with the sporadic form being the most prevalent. The disease was first documented by Hans Creutzfeldt in 1920 and subsequently by Alfons Jakob in 1921 and 1923^3^. Etiologically, CJD is caused by a prion, a discovery made by Stanley Prusiner in 1960, who later received the Nobel Prize for his work^4^^,^^5^. The disease-causing prion protein undergoes conformational changes, leading to alterations in the previously normal cellular prion protein^5^. The pathological manifestations observed in CJD consist of vacuolar lesions that give the brain a spongiform appearance, particularly affecting the basal ganglia, thalamus, cerebellum, and cerebral cortex^3^.

Although HIV infection can affect the central nervous system and cause various neurological complications, prion diseases are extremely rare and present a complex pathological scenario with significant diagnostic challenges^6^^,^^7^. Here, we report a rare case of a person living with HIV who developed a severe neurological condition that was ultimately diagnosed as Creutzfeldt-Jakob Disease (CJD).

## Case report

**Fig. 1:**
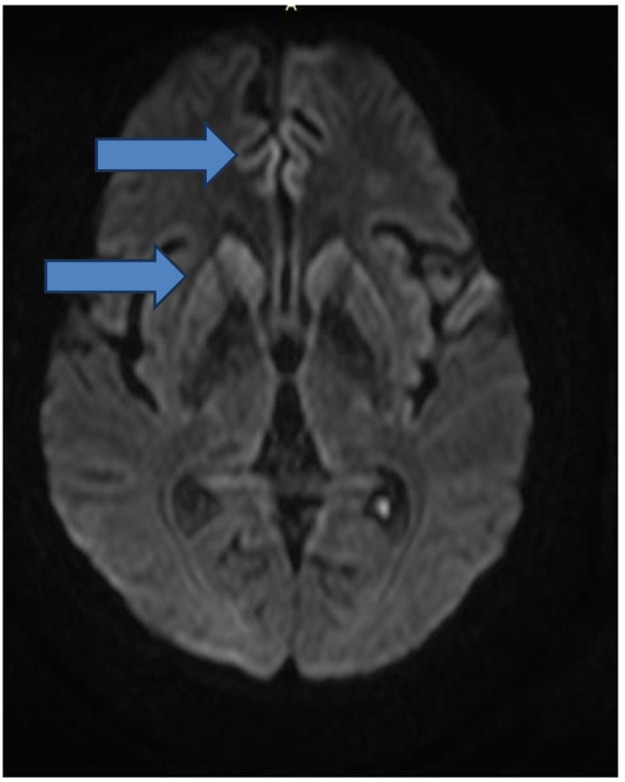
The brain MRI diffusion sequence images show a symmetric and bilateral hypersignal in the caudate and putamen, as well as in the superior frontal gyri (blue arrow).

**Fig. 2:**
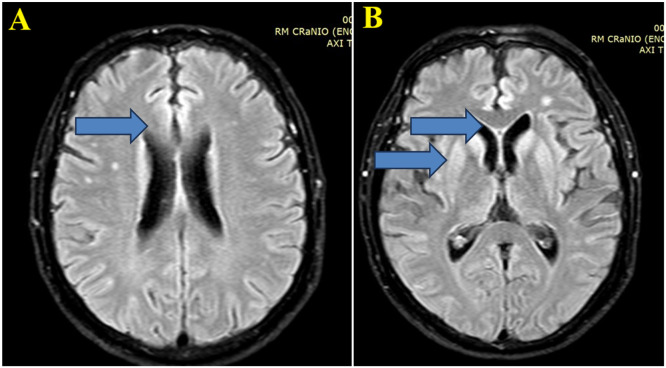
(A) Brain MRI images with Axial FLAIR sequence at the centrum semiovale show a small cortical hypersignal in the superior frontal gyrus bilaterally (blue arrow). (B) Brain MRI images at the basal ganglia level also demonstrate a symmetric and bilateral hypersignal in the caudate and putamen, as well as in the superior frontal gyri (blue arrow).

A 59-year-old man was diagnosed with HIV associated with Pulmonary Tuberculosis (TB) in late 2012 (at age 48) through ELISA and Western Blot serological tests, as well as a sputum culture for Acid-Fast Bacilli (AFB). He initially received treatment for TB, followed by Antiretroviral Therapy (ART) two weeks later, consisting of tenofovir (TDF), lamivudine (3TC), and efavirenz (EFV). Laboratory tests before starting ART showed a CD4 count of 18 cells/mcL (4 %) and a CD8 count of 203 cells/mcL (45 %), with a Viral Load (VL) of 224,906 copies/mL (Log=5.35). After completing TB treatment, his CD4 count increased to 167 cells/mcL (17.9 %), and his VL decreased to 82 copies/mL (Log=1.91). The patient developed Immune Reconstitution Inflammatory Syndrome (IRIS) with cervical lymphadenitis, which was managed with aspiration, and experienced persistent peripheral neuropathy during TB treatment, requiring pyridoxine supplementation. During this period, he also developed systemic arterial hypertension, type 2 diabetes mellitus, dyslipidemia, and keratitis related to past pterygium surgery. After 11-years of regular antiretroviral therapy, which was later switched to lamivudine and dolutegravir, he maintained persistent undetectable HIV viremia and adequate pharmacological control of systemic arterial hypertension, type 2 diabetes and hypercholesterolemia, using losartan 50 mg/day, metformin 1g/day, and atorvastatin 10 mg/day. He persistently reported bilateral 'boot distribution' paresthesia, attributed to an adverse reaction to isoniazid. During a routine follow-up on May 8, 2023, he mentioned that his partner had been complaining about him forgetting personal items, such as his wallet and car keys, despite the patient’s own disagreement. At this time the International HIV Dementia Scale scored seven points, and a brain MRI was recommended. Unfortunately, he did not undergo the requested brain MRI and returned only three months later, experiencing a sudden worsening of symptoms, including increased neuropathy, weight loss, social withdrawal, and somnolence. Neurological examination revealed temporal disorientation, ataxia, a left Babinski sign, hyperreflexia in the right patellar tendon, exhausted clonus in the right foot, and dysdiadochokinesia. Myoclonus was not present. Hospitalization was recommended, when evaluated by a neurologist, and MRI (in August 9) showed a restricted diffusion hypersignal located bilaterally in the insula, basal ganglia, and anterior cingulate cortex (Fig. 1). Seventeen days after this first and brief hospitalization, MRI showed symmetric and bilateral hypersignal in the caudate and putamen, as well as in the superior frontal gyri (Fig. 2). Concurrent laboratory tests showed no abnormalities, including a non-reactive VDRL and normal renal, thyroid, and liver function. A lumbar puncture was also performed, and partial analysis of the Cerebrospinal Fluid (CSF) revealed normal cell counts and glucose and protein levels. Tests for pyogenic bacteria, fungi, and tuberculosis and PCR for herpes simplex virus types 1 and 2 were all negative. RNA PCR for HIV in the CSF was also negative. In addition, CSF autoantibodies for autoimmune and paraneoplastic encephalitis and 14-3-3 protein analyses were requested. At this point, we suspected rapidly progressive dementia, with prion disease and immune-mediated encephalitis considered as the main possibilities. Due to the delay in obtaining the 14-3-3 protein results, the patient was hospitalized for pulse therapy with methylprednisolone (1.0 g/day for 5-days), followed by intravenous immunoglobulin (IVIG – 2.0 g/kg for 5-days). During the second hospitalization (lasted 56-days), repeat MRI showed the same abnormalities described above, but the patient’s condition worsened, with further memory decline, spastic tetraparesis, hypophonia, and bradykinesia in both the upper and lower limbs. Furthermore EEG video was performed due to its longer duration in an attempt to capture changes characteristic of CJD, such as triphasic waves. The disease progression was rapid, leading to a state of akinetic mutism. A Gastrostomy Tube (GTT) was placed due to dysphagia. The patient developed a fever caused by aspiration pneumonia, which was treated with antibiotics, and was discharged with the GTT, spastic tetraparesis and akinetic mutism. After 60-days, the 14-3-3 protein results were received, revealing high titers, and the family was consulted about initiating palliative care to avoid invasive measures. During his course, the patient underwent 3 EEGs, all of which revealed only nonspecific slowing. RT-QuIC analysis of the CSF was positive. He passed away 6-months after the onset of neurological symptoms.

## Discussion

The annual incidence of Creutzfeldt-Jakob Disease (CJD) is approximately 1 case per million individuals, making it a rare and incurable condition^8^. Such rarity becomes particularly evident in specialized dementia services, where the etiology of rapidly progressive dementias is assessed^9^. The main point of this case is the extremely rare association of prion disease with HIV infection^6^. To the best of our knowledge, fewer than 10 cases have been diagnosed worldwide, with only 3 cases reported in Brazil^10^^,^^11^.

Sporadic CJD, the most common form of the disease, manifests with cognitive, visual, cerebellar, and motor (pyramidal/extrapyramidal) signs and symptoms, all of which are integral to the current diagnostic criteria^12^.

Alongside the clinical presentation, complementary examinations play a key role in the diagnosis of CJD^3^^,^^13^. Brain MRI typically reveals lesions characterized by Diffusion-Weighted Imaging (DWI) hyperintensities and signal abnormalities on FLAIR and T2-weighted sequences, particularly in the cerebral cortex and basal ganglia, as observed in the present case^14^ .Electroencephalogram findings, though limited in sensitivity, may show triphasic waves or periodic complexes, which can contribute to the diagnosis^15^. Cerebrospinal fluid analysis can help detect elevated levels of proteins such as 14-3-3, tau, and p-tau, which are associated with the disease^16^. Furthermore, the identification of pathological prion protein through RT-QuIC in nasal mucosa or CSF is notable for its high specificity^17^.

The present case highlights the importance of a thorough investigation in cases of rapidly progressive dementia in HIV-infected patients. This investigation should include screening for autoimmune encephalitis and central nervous system infections, as well as metabolic and demyelinating conditions. In our patient, the clinical course following immunotherapy, CSF examination (including RT-QuIC), laboratory tests, systemic neoplasm screening, and brain MRI confirmed CJD while ruling out CNS infections, metabolic diseases, and autoimmune and paraneoplastic encephalitis. The ability to perform RT-QuIC in suspected cases is crucial, given its high specificity in diagnosing prion diseases. Additionally, the investigation of autoimmune and paraneoplastic encephalitis and CNS infections in CSF is essential.

In conclusion, despite the lack of a well-established relationship between HIV infection and CJD, this rare condition should still be considered, especially in patients with controlled viral loads and adequate immune reconstitution who rapidly progress to a state of dementia.

## Conflicts of interest

The authors declare no conflicts of interest.
